# The Use of the Term Virtual Reality in Post-Stroke Rehabilitation: A Scoping Review and Commentary

**DOI:** 10.5334/pb.1033

**Published:** 2021-06-03

**Authors:** Hanne Huygelier, Emily Mattheus, Vero Vanden Abeele, Raymond van Ee, Céline R. Gillebert

**Affiliations:** 1 Brain and Cognition, KU Leuven, Leuven, Belgium; 2 e-Media | Human-Computer Interaction, KU Leuven, Leuven, Belgium; 3 Donders Institute for Brain, Cognition and Behavior, Radboud University, Nijmegen, The Netherlands; 4 Philips Research, High tech Campus, Eindhoven, The Netherlands; 5 TRACE, Ziekenhuis Oost-Limburg, Genk, Belgium

**Keywords:** Virtual reality, stroke, neurorehabilitation, rehabilitation, review

## Abstract

Virtual reality (VR) offers many opportunities for post-stroke rehabilitation. However, “VR” can refer to several types of computer-based rehabilitation systems. Since these systems may impact the feasibility and the efficacy of VR interventions, consistent terminology is important. In this study, we aimed to optimize the terminology for VR-based post-stroke rehabilitation by assessing whether and how review papers on this topic defined VR and what types of mixed reality systems were discussed. In addition, this review can inspire the use of consistent terminology for other researchers working with VR. We assessed the use of the term VR in review papers on post-stroke rehabilitation extracted from Scopus, Web of Science and PubMed. We also developed a taxonomy distinguishing 16 mixed reality systems based on three factors: immersive versus semi-immersive displays, the way in which real and virtual information is mixed, and the main input device. 64% of the included review papers (N = 121) explicitly defined VR and 33% of them described different subtypes of VR, with immersive and non-immersive VR as the most common distinction. The most frequently discussed input devices were motion-capture cameras and handheld devices, while regular 2D monitors were the most frequently mentioned output devices. Our analysis revealed that reviews on post-stroke VR rehabilitation did not or only broadly defined “VR” and did not focus on a specific system. Since the efficacy and feasibility of rehabilitation may depend on the specific system, we propose a new data-driven taxonomy to distinguish different systems, which is expected to facilitate communication amongst researchers and clinicians working with virtual reality.

## Introduction

Stroke is a leading cause of disability worldwide, necessitating effective rehabilitation strategies (Institute for Health Metrics and Evaluation (IHME), 2018). It can have a severe and persistent impact on patients with respect to sensorimotor, cognitive and perceptual functions (Edmans & Lincoln, 1987; Hochstenbach et al., 2005; Kauhanen et al., 2000; McDowd et al., 2003; Nys et al., 2007; Pearce et al., 2016; Sue-Min et al., 2002). In the motor domain, patients can have long-lasting impairments affecting their upper-limbs, gait and balance (Hesse & Werner, 2003; Kao et al., 2014; Langhorne et al., 2009; Raghavan, 2015; Sibley et al., 2009; Verheyden et al., 2008). In the cognitive domain, patients can experience difficulties in language, attention, executive functions, praxis and memory (Douiri et al., 2013; Jaillard et al., 2009; Kauhanen et al., 2000; Makin et al., 2013; McDowd et al., 2003; Nys et al., 2007). Stroke can also lead to apathy and depression (Espárrago et al., 2015; Mayo et al., 2009; Nelson et al., 1993), fatigue (Christensen et al., 2008; Snaphaan et al., 2011) and has a negative impact on activities of daily living (Sue-Min et al., 2002) and return to work (Katie et al., 2009).

### Virtual reality offers opportunities for post-stroke rehabilitation

There are many challenges in post-stroke rehabilitation. For instance, research investigating different therapies (i.e., physical, occupational, perceptual training) has consistently shown that functional outcome is better when therapy doses are higher (Lohse et al., 2014; Ong et al., 2012; Parker et al., 2013). However, it is not straightforward to deliver high therapy doses in clinical practice (Bernhardt et al., 2007; Lang et al., 2009; Ong et al., 2012; Otterman et al., 2012). Furthermore, treatment adherence to physical therapy of stroke patients is often limited (Langhorne et al., 2009; Miller et al., 2017; Tiedemann et al., 2012). The latter is especially the case when the therapy is experienced to be boring (Miller et al., 2017). Although most research has focused on occupational and physical rehabilitation, there is also evidence that treatment adherence to cognitive rehabilitation can be problematic (Wentink et al., 2018). Finally, it is challenging to develop ecologically valid tasks for cognitive rehabilitation, which may explain why training effects do not transfer to daily life activities (Rizzo et al., 2004).

*Virtual reality (VR)* has the opportunity to tackle some of these challenges. For instance, it can provide real-time multisensory feedback, task variation, objective progression and task-oriented repetitive training (Laver et al., 2012; Porras et al., 2018; Rizzo et al., 2004). It can also improve the precision of performance measurements and the standardization of treatment protocols (Bohil et al., 2011; Iosa et al., 2012; Massetti et al., 2018; Rizzo et al., 2004; Rose et al., 2005; Tieri et al., 2018). Furthermore, it can have positive effects on motivation. For example, stroke patients reported less fatigue when using a robotic device to navigate a virtual plane displayed on a regular computer monitor than without this visual feedback (Mirelman et al., 2009).

However, the use of virtual reality in post-stroke rehabilitation also faces several challenges. A first challenge is cybersickness. Previous research has revealed that VR applications that create a high sense of presence in the virtual environment also induce more cybersickness (Weech et al., 2019). Other design characteristics of the VR application (Davis et al., 2015; Porcino et al., 2017; Stanney & Hash, 1998) and of the end-users (Arns & Cerney, 2005) can also impact the prevalence of cybersickness. Thus, although current VR games can be designed in ways that effectively minimizes cybersickness in certain populations (Appel et al., 2020; Huygelier et al., 2019; Plechatá et al., 2019), it remains necessary to assess cybersickness for each VR application and end-user group. Second, the potential of VR to increase treatment dosage in post-stroke rehabilitation depends on the ability of patients to use VR systems independently. Although some research has investigated the safety, user experience and usability of the latest generation of VR systems in stroke patients (Huygelier et al., 2020; Lee et al., 2020; Spreij et al., 2020; Weber et al., 2019), none of these studies addressed the feasibility of independent use by patients. Third, although virtual reality offers many opportunities for rehabilitation, most studies did not address the added value nor cost-effectiveness of VR rehabilitation relative to other therapies.

### The use of “virtual reality” in post-stroke rehabilitation

In 1994 Milgram et al. (p. 1) (Milgram & Kishino, 1994) defined a *VR environment* as “*an environment in which the participant-observer is totally immersed in, and able to interact with, a completely synthetic world*.” Since then, many authors consider the level of *immersion* as an objective property of the technological system (Bohil et al., 2011; Milgram & Kishino, 1994; Slater, 2003), which depends on the intensity and fidelity of the sensory stimulation provided by that system (Bohil et al., 2011; Milgram & Kishino, 1994; Slater, 2003). It can be distinguished from *presence*, which refers to the subjective experience of being in the virtual environment without being aware of the technological mediation (Bohil et al., 2011). More immersive technology has a higher chance to create experiences that result in a high sense of presence, but the immersive nature of the technology does not guarantee a high sense of presence. Indeed, in contrast to immersion being related to the technology, sense of presence can vary between individuals. For instance, one study reported that stroke patients reported less sense of presence than healthy individuals, although they used the same immersive VR system (Borrego et al., 2019). In addition, Milgram distinguished several *mixed reality* systems based on the extent to which real and virtual information were mixed (Milgram et al., 1995; Milgram & Kishino, 1994). *Augmented reality (AR)* refers to systems in which virtual information is superimposed over the real environment (e.g., Pokémon Go), while in *augmented virtuality (AVR)* real world information is superimposed over a virtual world, and in VR *all* information is virtual. Thus, systems in which a virtual avatar represents movements by the user are considered VR, while systems in which an image of the user is added onto a virtual environment are considered AVR.

Although older definitions of VR emphasized its immersive nature and distinguished VR from other categories of mixed reality (Milgram & Kishino, 1994; Steuer, 1992), “virtual reality” has been ill-defined in the literature on post-stroke rehabilitation (Garrett et al., 2018; Perez-Marcos, 2018; Tieri et al., 2018). “Virtual reality” has been used to refer to several types of computerized rehabilitation, ranging from less immersive systems that display 3D environments on regular 2D monitors to more immersive systems that use head mounted displays (HMD) that offer a near full field of view (Huygelier et al., 2019; Iosa et al., 2012; Perez-Marcos, 2018; Tieri et al., 2018). Furthermore, little attention has been given to the dependence of the efficacy and feasibility of VR rehabilitation on the specific system (Garrett et al., 2018). Some of the advantages of VR for stroke rehabilitation may indeed only be characteristic of immersive VR. For instance, immersive VR may increase motivation for rehabilitation tasks, potentially leading to higher therapy doses (Massetti et al., 2018; Tieri et al., 2018). It can also act as an enriched environment with beneficial effects on neuroplasticity (Laver et al., 2012). Furthermore, it allows one to create ecologically valid activities such as car driving, which may in turn improve transfer of rehabilitation effects to daily life (Rizzo & Kim, 2005; Rizzo et al., 2004). Finally, immersive VR allows one to navigate in 3D space, thereby creating opportunities for the rehabilitation of spatial cognition impairments, such as hemispatial neglect (Aravind & Lamontagne, 2014; Dvorkin et al., 2012; Myers & Bierig, 2000; Pedroli et al., 2015).

In summary, the lack of clarity in the use of the term VR has made it difficult to navigate the literature on post-stroke rehabilitation and infer the efficacy and feasibility of specific rehabilitation systems.

### The current study

The purpose of this study was to gain insight in the use of the term “VR” in review papers discussing VR post-stroke rehabilitation and to provide new terminology to describe rehabilitation systems. To this end, we extracted the definitions used for VR and which type of mixed reality system was referred to as VR. In addition, we assessed whether different systems were used for the rehabilitation of specific functional impairments and whether immersive VR was more frequently mentioned over time.

## Method

We performed a scoping review following the PRISMA guidelines. PubMed, Web of Science Core collection and Scopus were searched for published reviews on VR post-stroke rehabilitation. Publications from the inception of the databases until and including the 29^th^ of July 2019 were searched. The keywords were “stroke”, “virtual reality” and “rehabilitation” and synonyms for these terms (***Table 1***). As we aimed to establish how the term “virtual reality” has been defined and to which systems it has referred, we did not search papers mentioning other types of computerized rehabilitation (e.g., augmented reality, serious games).

**Table 1:** Search strings used in different databases.


DATABASE	SEARCH SYNTAX		N

PubMed	((stroke*[Title/Abstract] OR cva*[Title/Abstract] OR poststroke[Title/Abstract] OR post-stroke[Title/Abstract] OR apoplex*[Title/Abstract]) OR ((brain[Title/Abstract] OR cerebell*[Title/Abstract] OR intracran*[Title/Abstract] OR intracerebral[Title/Abstract] OR vertebrobasilar[Title/Abstract]) AND (haemorrhag*[Title/Abstract] OR hemorrhag*[Title/Abstract] OR ischemi*[Title/Abstract] OR ischaemi*[Title/Abstract] OR infarct*[Title/Abstract] OR haematoma*[Title/Abstract] OR hematoma*[Title/Abstract] OR bleed*[Title/Abstract])) AND (rehabilit*[Title/Abstract] OR neurorehabilit*[Title/Abstract] OR treat*[Title/Abstract] OR heal*[Title/Abstract] OR restor*[Title/Abstract] OR cur*[Title/Abstract] OR improve*[Title/Abstract] OR recov*[Title/Abstract]) AND (virtual reality[Title/Abstract] OR Oculus Rift[All Fields] OR HTC Vive[All Fields] OR immersive[Title/Abstract] OR virtual environment[Title/Abstract]) AND (Review[Filter])		100

Web of Science	TS = ((stroke OR cva* OR poststroke OR post-stroke OR apoplex*) OR ((brain OR cerebell* OR intracran* OR intracerebral OR vertebrobasilar) AND (haemorrhag* OR hemorrhag* OR ischemi* OR ischaemi* OR infarct* OR haematoma* OR hematoma* OR bleed* OR damage)))	#1	302

	TS = (rehabilit* OR neurorehabilit* OR treat* OR heal* OR restor* OR cur* OR improve* OR recov*)	#2	

	TS = (virtual reality OR Oculus Rift OR HTC Vive OR immersive OR virtual environment)	#3	

	TS = (systematic AND review OR review OR meta-analysis OR literature AND search)	#4	

	(#4 AND #3 AND #2 AND #1) OR ((#3 AND #2 AND #1) *AND* **DOCUMENT TYPES:** (Review))		

Scopus	TITLE-ABS-KEY (virtual AND reality) AND TITLE-ABS-KEY (rehabilit*) AND TITLE-ABS-KEY (stroke) AND (LIMIT-TO (DOCTYPE, “re”))		121


*Note*: *N* = number of search results. We used the search string for “stroke” developed by Veerbeek et al. (2014). The search string for “rehabilitation” was iteratively optimized and the search string for “virtual reality” was restricted as we only wanted to include reviews that use the term “virtual reality”.

**Figure 1:**
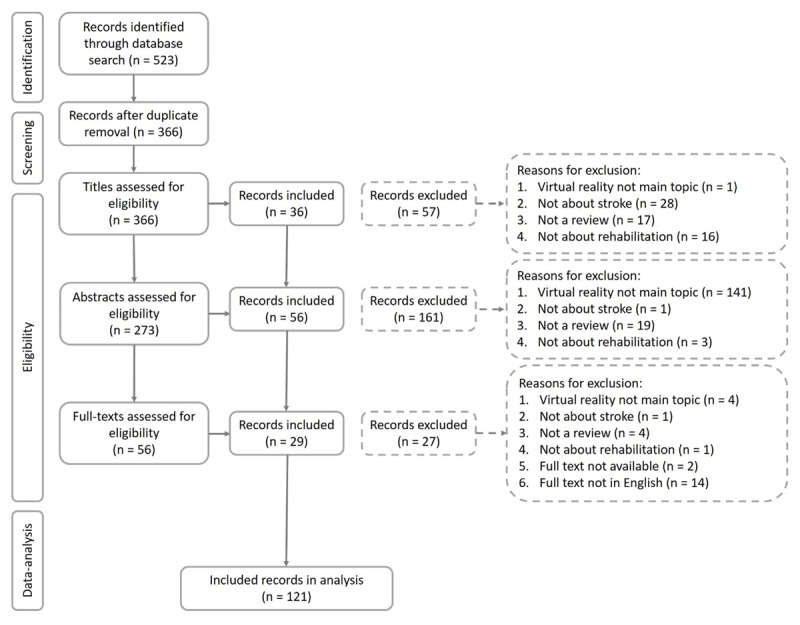
Flow chart of in- and exclusion of reviews.

Reviews that discussed stroke patients or multiple patient groups including stroke patients were included. Reviews that mentioned multiple rehabilitation methods including VR treatments were included. Reviews that solely discussed other patient groups (e.g., Parkinson’s disease) or other rehabilitation methods without referring to them as “VR” (e.g., treadmill training) were excluded. Articles that presented previously unpublished data, editorials, study protocols and commentaries of single articles were excluded. Two raters (among which HH) independently evaluated the in- and exclusion criteria by subsequently screening the titles, the abstracts and the full texts (***Figure 1***). The two raters discussed disagreements (22% of all records) to reach a unanimous decision.

### Data-extraction and data-analysis

#### Description of included reviews

We coded the main topic of the review, distinguishing reviews that either discussed efficacy, feasibility, design of VR therapies, or other topics (e.g., theoretical, methodological reviews and broad reviews without a specific focus). Moreover, we documented whether the review solely discussed VR rehabilitation *versus* multiple rehabilitation methods including VR. Additionally, we documented whether the review discussed a general patient group including stroke (i.e., general health, neurological disorders, acquired brain injury) or stroke patients only. We also documented which functional impairment the review discussed distinguishing upper or lower limb motor impairments, activities of daily living (ADL), memory, hemispatial neglect and/or pain perception. If reviews discussed multiple outcomes, we coded the functional impairments either as multiple domains (i.e., motor and cognition or motor and activities and participation), multiple motor impairments or multiple cognitive impairments.

To better understand the topics covered by reviews we performed a network and hierarchical cluster analysis. Each review characteristic was coded as present or absent per review. Then, we calculated the frequencies of each of these features and the frequency of co-occurrences of each pair of features using the R network package v1.15 (Butts et al., 2019; R Core Team, 2016). We used an undirected network with the weights based on the frequencies. The network was visualized with the ggnetwork package in R using the circular layout algorithm (Briatte, 2016). To categorize our reviews we used a hierarchical cluster analysis based on a binary distance measure from the stats R package (R Core Team, 2016) and visualized the results using the ggtree package in R (Yu et al., 2017).

#### How was VR defined?

First, we checked whether the term “virtual reality” was defined in the full texts of the reviews. Based on all reviews that defined VR, we developed a list of terms used in the definitions and then coded whether each term occurred in a definition. We used an undirected network analysis with weights based on the frequencies to find the terms used to define VR that co-occurred most often in reviews. We also documented whether authors distinguished different types of VR, how many subtypes were distinguished and how they were labelled.

#### Which technological systems, input- and output devices were considered VR?

Reviews sometimes referred to a single input- (i.e., the device that is used by the user to provide input to the system) or output device (i.e., the device that is used to provide input to the user by the system), or a technological system, which is a combination of input and output devices (e.g., IREX GestureTek). We documented the input and output devices and systems that were mentioned in the reviews. Descriptions of input and output devices can be found in Table S1 and Table S2. Only devices that were mentioned as examples of what the authors viewed as VR were included. If a review discussed assessment and rehabilitation, only rehabilitation systems were included. If a technological system was mentioned rather than specific devices, the devices were inferred from the system. For instance, if Nintendo Wii Sports was mentioned, we inferred that a regular 2D monitor and handheld motion controller were used. If the description of a system was not specific enough to be classified (e.g., “VR System”), we searched the cited articles for more information.

## Results

A total of 366 unique records were identified, of which 121 met the inclusion criteria (***Figure 1***). To better understand the topics covered by reviews, we performed a network and hierarchical cluster analysis, which are techniques that are increasingly used to analyse and report results of systematic reviews (Ananiadou et al., 2009; Thomas et al., 2011).

### Description of reviews

**Figure 2:**
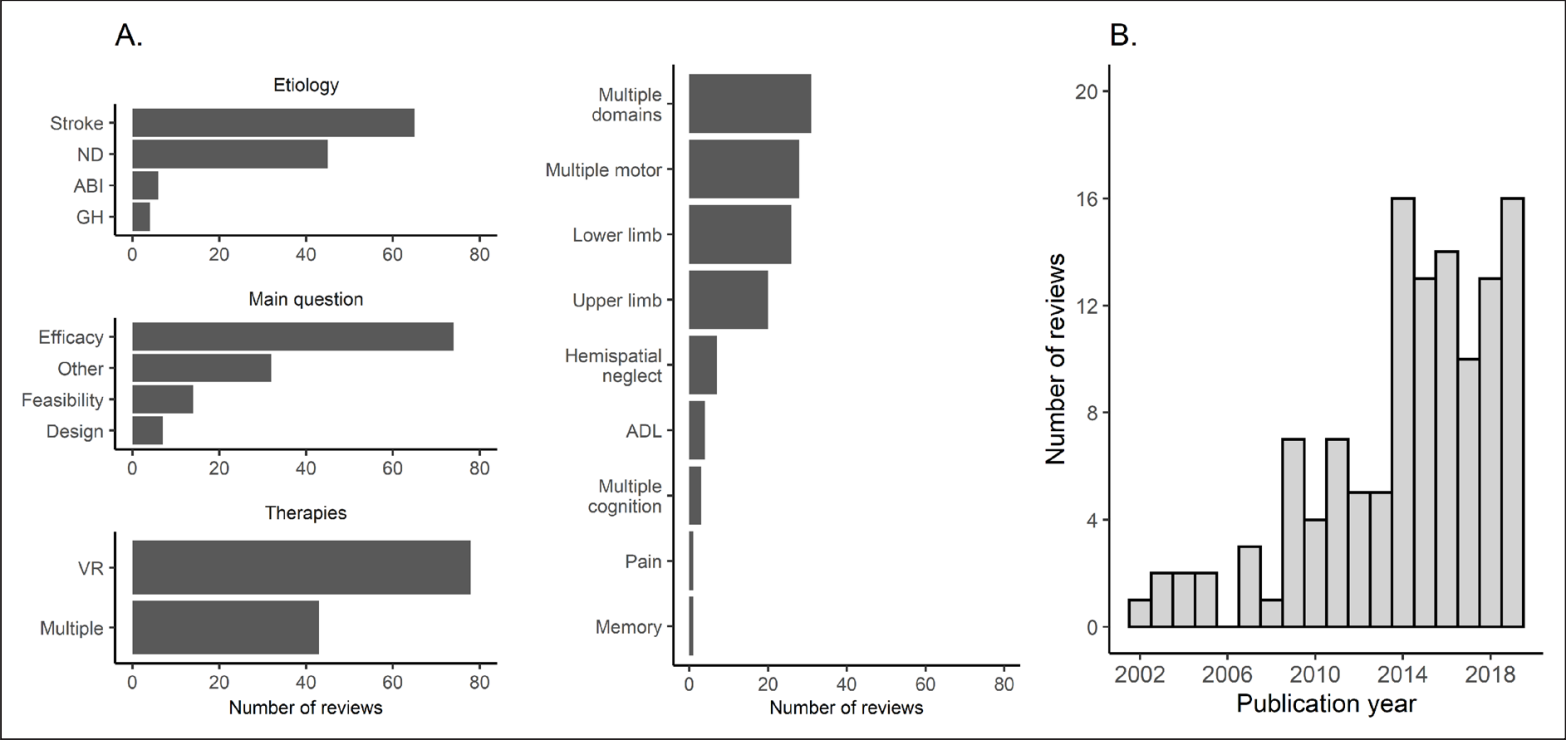
Review characteristics. **Panel A** represents the number of reviews for different review characteristics. **Panel B** depicts the number of reviews per publication year. GH = general health conditions, ND = neurological disorders, ABI = acquired brain injury, ADL = activities of daily living.

**Figure 3:**
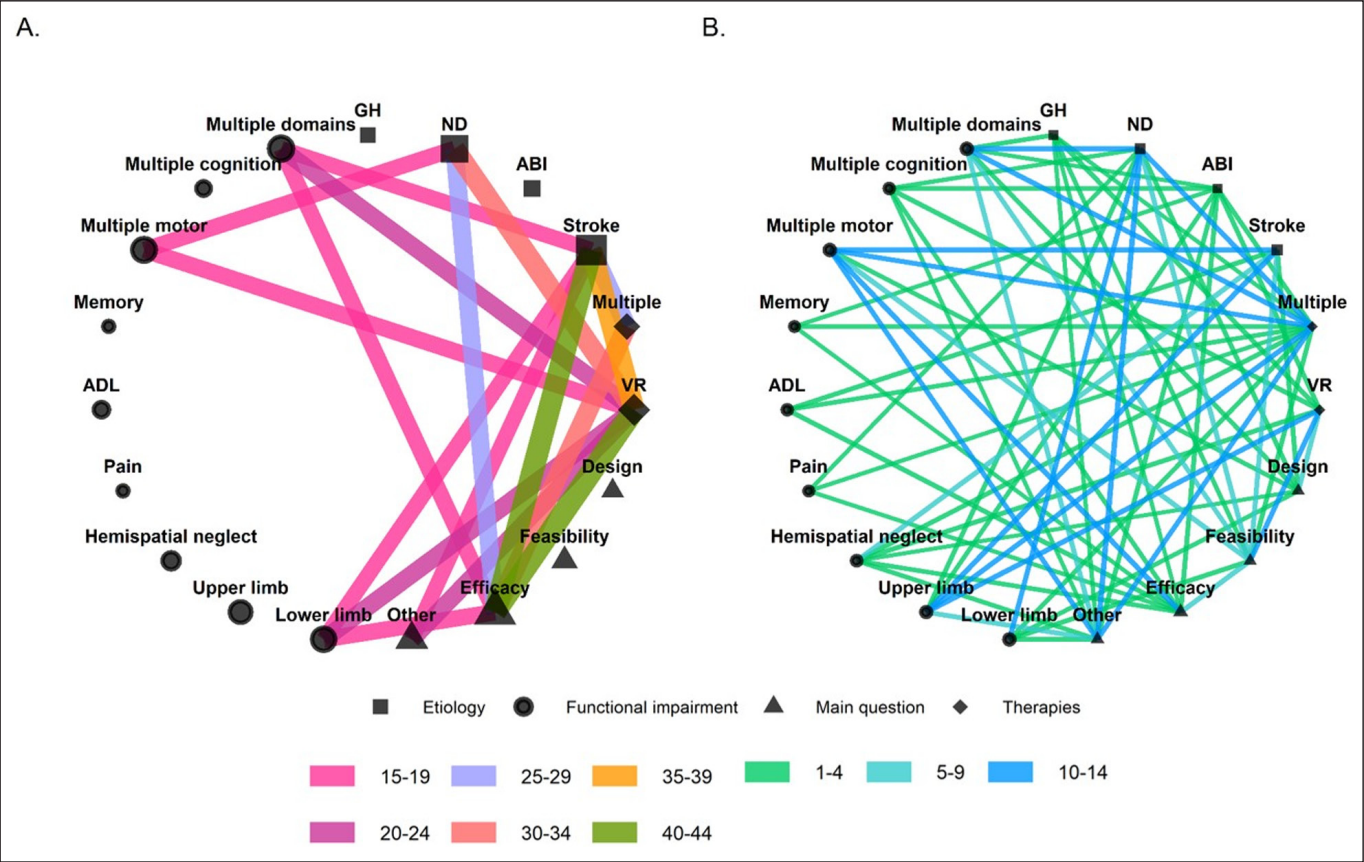
**Panel A** represents the strongest and **Panel B** the weakest connections in the network of review characteristics. Colors represent the number of times that two features co-occurred across the included reviews. The width of the edges are scaled according to the number of reviews in which a pair of features co-occurred. The size of the nodes is scaled according to the number of reviews in which the feature occurred. GH = general health conditions, ND = neurological disorders, ABI = acquired brain injury, ADL = activities of daily living.

54% of reviews discussed stroke, 37% discussed neurological disorders, 5% discussed acquired brain injury and 3% discussed general health conditions (***Figure 2A***). 26% reviews discussed multiple functional domains, while 61% discussed motor impairments, either multiple motor impairments (23%) or upper- (17%) or lower limb impairments (22%) (***Figure 3A***). 7% of reviews discussed cognitive or perceptual impairments and 3% discussed activities of daily living (ADL). 61% reviews discussed efficacy/effectiveness, while only 12% discussed feasibility of therapies (***Figure 2A***). The majority of reviews (65%) discussed what they labelled as VR therapies, while the other reviews discussed multiple rehabilitation methods including VR (***Figure 3A***). The reviews were published between 2002 to 2019 (M = 2014, SD = 4) with 50% of reviews published since 2015 (***Figure 2B***).

The most frequently connected review characteristics included efficacy, VR rehabilitation or multiple rehabilitation methods, multiple motor impairments or lower-limb motor impairments and stroke or neurological disorders in general (***Figure 3***).

**Figure 4:**
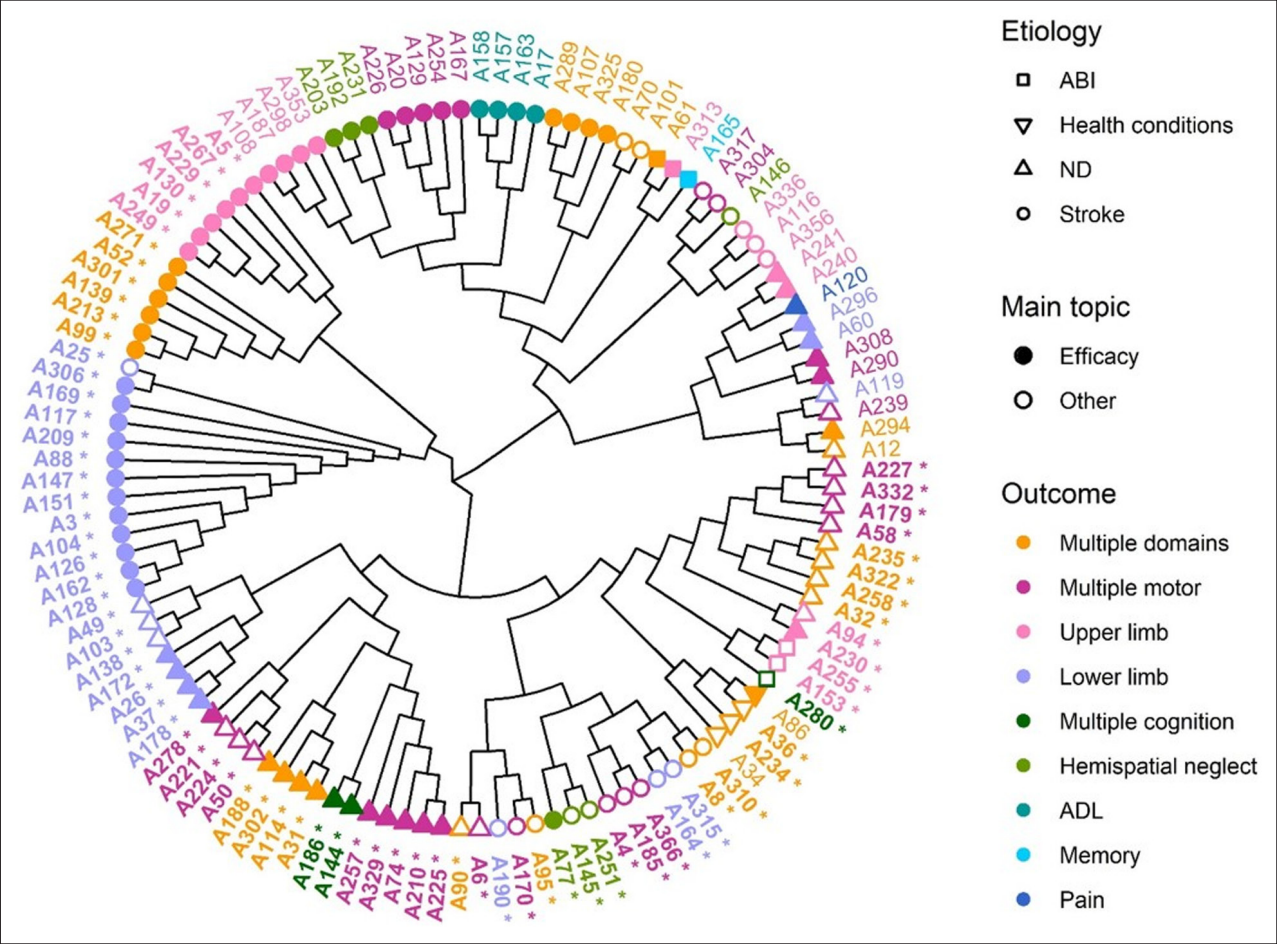
Hierarchical clustering of reviews based on aetiology, functional impairment, the main question of the review and whether the review discussed VR or multiple rehabilitation methods including VR. A list of references for each article ID is available online (*https://doi.org/10.6084/m9.figshare.11902614.v1*). Labels in bold font with a * represent reviews that focused on VR rehabilitation, while labels in plain font represent reviews that focused on multiple rehabilitation methods including VR. ND = neurological disorders, ABI = acquired brain injury, ADL = activities of daily living.

The hierarchical cluster map in ***Figure 4*** conveys similar information as ***Figure 3***, but can guide readers to find reviews with certain features. Reviews placed closer to each other are more similar than reviews placed further apart. For instance, four studies forming a cluster discussed the efficacy of multiple rehabilitation methods for ADL in stroke patients. Also, among the bigger clusters, one cluster consists of reviews about the efficacy of VR rehabilitation for lower limb impairments in stroke patients or neurological disorders.

### How was VR defined?

**Figure 5:**
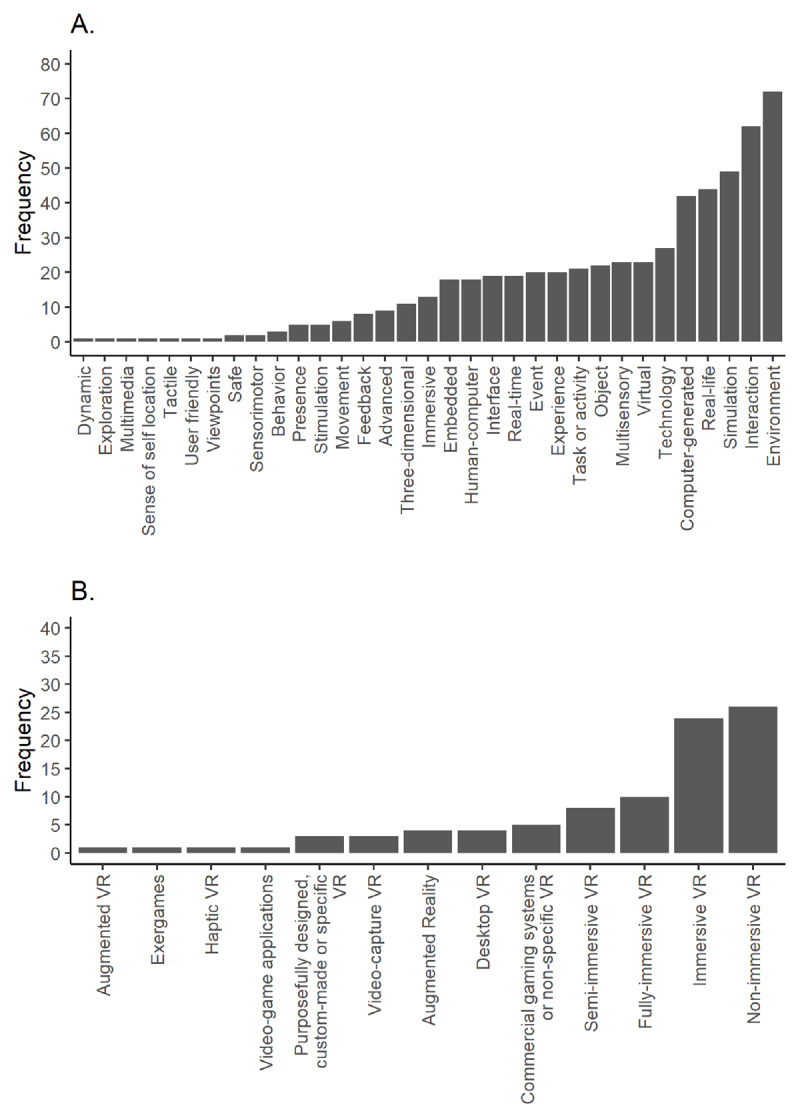
Terms used to define VR **(A)** and terms used to distinguish VR categories **(B)**.

**Figure 6:**
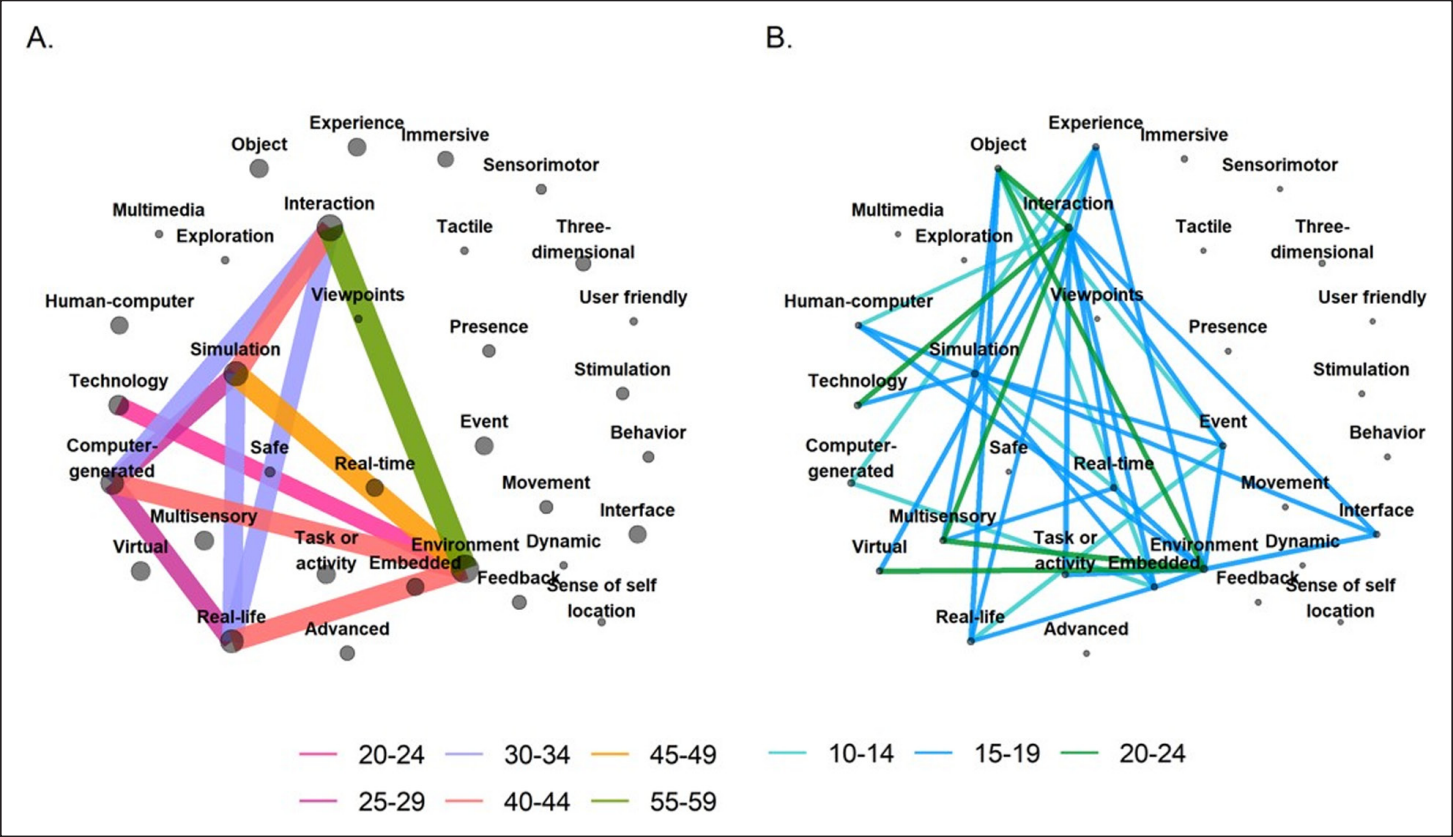
Network of terms used in VR definitions. **Panel A** represents the strongest connections and **panel B** represents the weakest connections in the network. The color represents the number of times that two features co-occurred across the included reviews. Connections smaller than 10 reviews are not visualized. The width of the edges are scaled according to the number of reviews in which a pair of features co-occurred. The size of the nodes is scaled according to the number of reviews in which the feature occurred.

64% of reviews defined “*virtual reality*”. In these reviews, the five most frequently used terms were an environment or world (92%), interaction (79%), simulation (63%), real-life or natural (56%) and computer-generated (54%) (***Figure 5A***). These terms co-occurred in more than 30% of these reviews (***Figure 6A***). 33% of reviews distinguished between different categories of VR. Non-immersive (65%) and immersive VR (60%) were the most common distinctions (***Figure 5B***).

### Which technological systems were considered VR?

85% of reviews mentioned technological systems or specific in- and output devices. We categorized the systems using Milgram’s taxonomy of mixed reality (Milgram et al., 1995; Milgram & Kishino, 1994). The first factor distinguished between VR, AR and AVR. The second dimension is the extent to which information is presented in an immersive way. Although immersion is best considered a continuum, we divided immersion in two categories. *Semi-immersive* systems (SIVR) use 2D displays with a limited field of view (i.e., *window on the world displays*) and the perspective on the environment does not change depending on head movements. *Immersive systems* (IVR) use head-mounted displays with head tracking or projections on large curved displays or rooms with multiple walls (i.e., CAVE) that almost provide a 360° field of view (Milgram & Kishino, 1994; Tieri et al., 2018).

**Figure 7:**
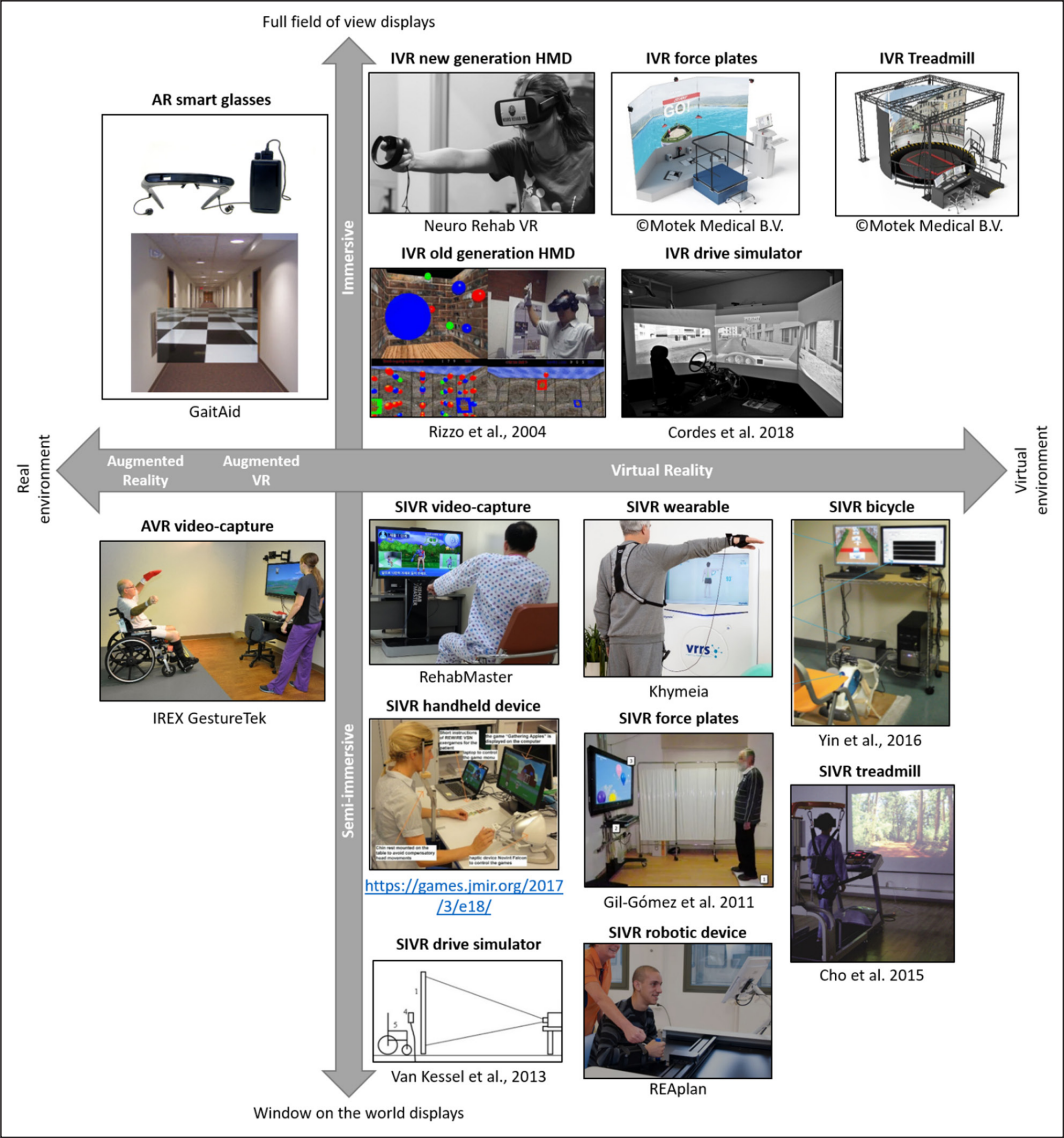
Taxonomy of mixed reality rehabilitation systems, based on the extent to which real and virtual information is mixed, the level of immersion and the main input device. AR = augmented reality, AVR = augmented virtual reality, IVR = immersive virtual reality, SIVR = semi-immersive virtual reality. HMD = head-mounted display. Permission to re-use images was provided by the original copyright owners in written format (Cho et al., 2015; Cordes et al., 2018; Gil-Gómez et al., 2011; Rizzo et al., 2004; Tobler-Ammann et al., 2017; van Kessel et al., 2013; Yin et al., 2016). The category SIVR desktop systems are not illustrated, because articles describing these systems typically do not include pictures of the hardware.

We also categorized systems based on the main input device. In the SIVR category, we distinguished systems that use a motion-capture camera (SIVR video-capture), a handheld device which could either be a motion-capture or haptic device (SIVR handheld device), a motion-capture wearable device (SIVR wearable device), balance or force plates (SIVR force plates), robotic devices (SIVR robotic device), a bicycle (SIVR bicycle), treadmill (SIVR treadmill), steering wheels and pedals (SIVR drive simulator), and a computer mouse or keyboard (SIVR desktop systems) (***Figure 7***). In the IVR category, we further subdivided systems based on the input and output devices. Some IVR systems used a treadmill either coupled with an HMD or a CAVE (IVR treadmill). Other IVR systems used a force plate as input device and CAVE or HMD (IVR force plates). There were also systems that combined an HMD or CAVE with a steering wheel and pedals (IVR drive simulators) and there were systems using the newest generation of HMDs with wide field of view and head-tracking (IVR new generation HMD) or older generation HMDs with a limited field of view (IVR old generation HMD). A spreadsheet with examples of systems for each of these categories is available online (*https://doi.org/10.6084/m9.figshare.11902614.v1*).

### Which technological systems were most frequently labelled as VR?

**Figure 8:**
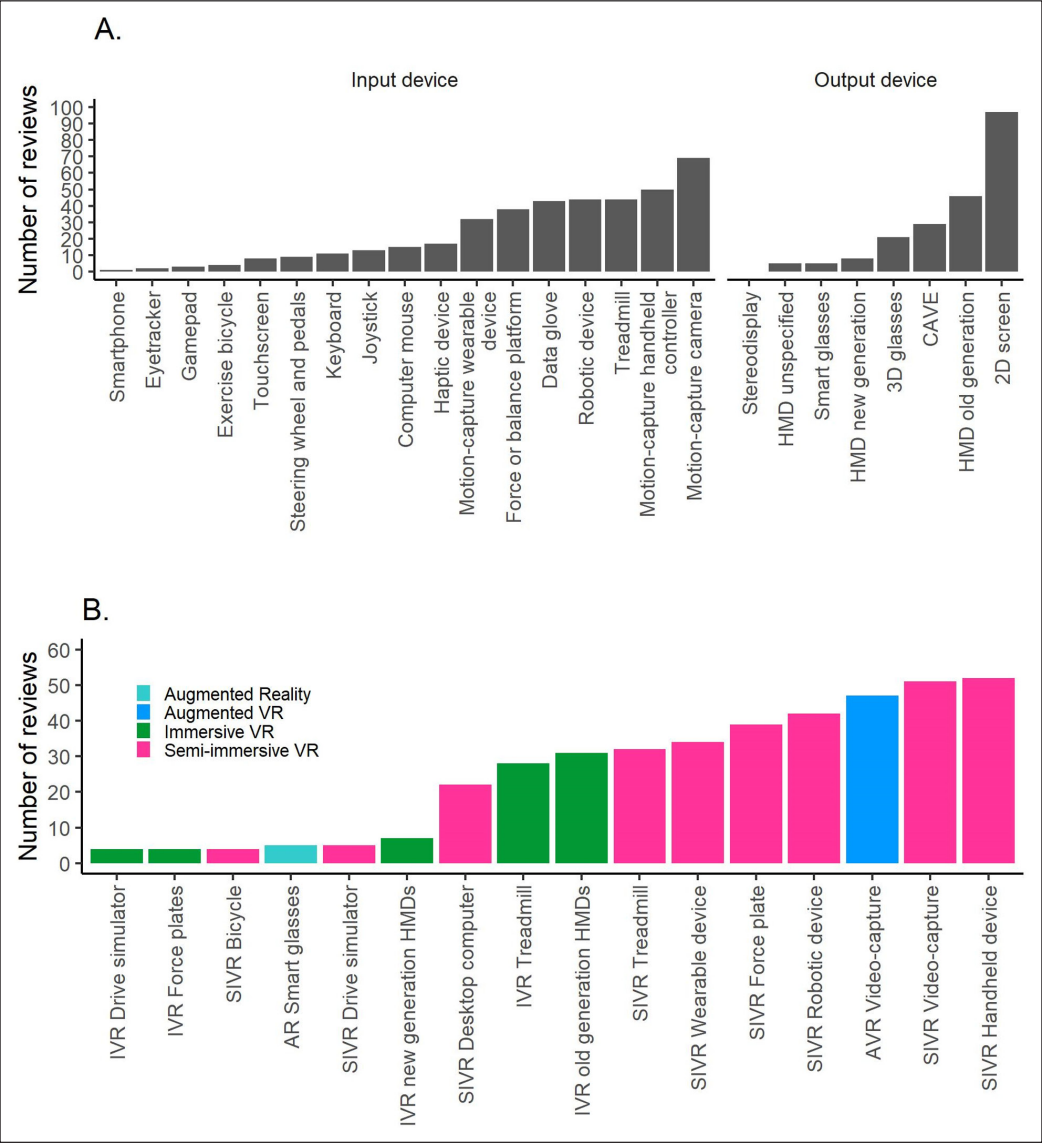
Frequency of different input and output devices **(A)** and mixed reality systems **(B)**.

The most frequently mentioned input devices were a motion-capture camera (67%) and motion-capture handheld device (49%). The two most popular output devices were a regular 2D monitor with a limited field of view (94%) and older generation HMDs (45%) (***Figure 8***). The most frequently mentioned mixed reality systems included *SIVR systems using handheld devices* (50%) and *video-capture* (50%), and *AVR video-capture* (46%). The next most popular systems were all SIVR in which patients provide input using a robotic device (41%), a balance/force plate (38%), or treadmill (31%). In the IVR category, systems using older generation HMDs with various interface devices (30%) or treadmill IVR systems (27%) were most frequently mentioned. The newest generation of HMDs were less frequently mentioned (7%).

Reviews mentioned on average 4 different mixed reality systems (SD = 2, Range: 1–10), and an average of 2 mixed reality subtypes (i.e., AVR, IVR, SIVR, AR) (SD = 0.8, Range: 1–3). There were 4 reviews that only mentioned IVR, 25 that only mentioned SIVR, 1 that only mentioned AVR and 1 that only mentioned AR systems. All other reviews mentioned multiple types of mixed reality.

### Differences in technological systems by functional impairment and publication year

**Figure 9:**
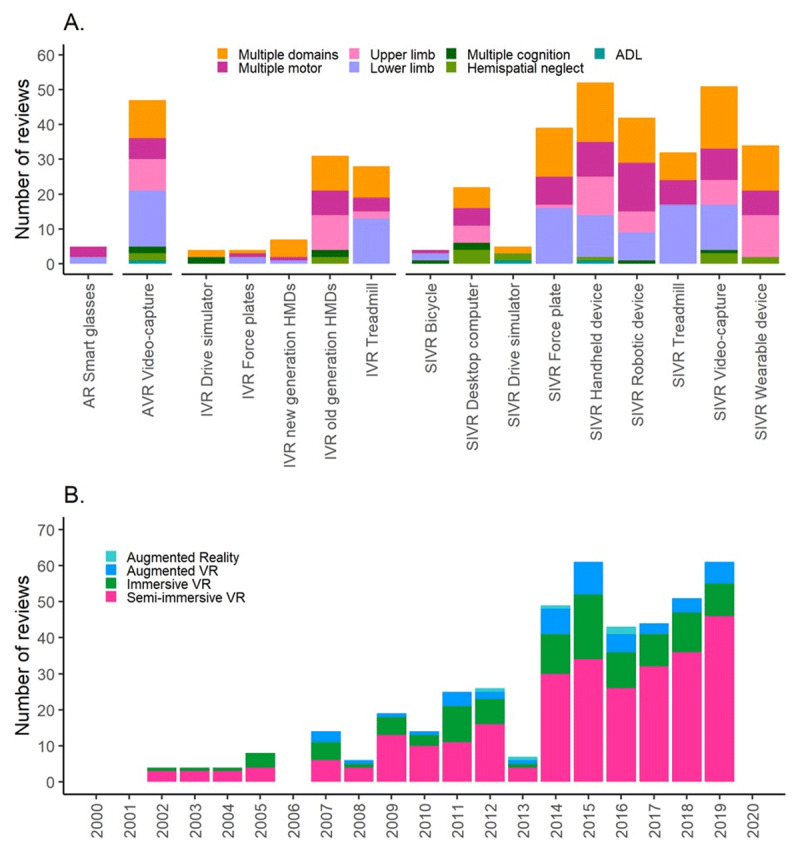
Frequencies of different functional impairments for each mixed reality system **(A)** and frequencies of mixed reality subtype for each publication year **(B)**.

We then assessed the number of times different mixed reality systems were discussed in relation to different functional impairments (***Figure 9A***) and publication year (***Figure 9B***). This analysis revealed that AR smart glasses were only discussed in the context of motor impairments, whereas AVR video-capture systems were discussed in relation to motor, cognitive impairments and ADL. SIVR and IVR systems using a treadmill, robotic device or force plate have mostly been discussed in the context of motor impairments, while IVR and SIVR drive simulators have mostly been discussed in relation to cognitive impairments or ADL. Finally, SIVR systems were the most frequently mentioned systems across publication years without a clear trend towards IVR systems (***Figure 9B***).

## Discussion

Our study revealed that VR was often not or only vaguely defined in review papers on VR post-stroke rehabilitation, confirming the lack of clarity in the use of VR terminology (Garrett et al., 2018; Perez-Marcos, 2018; Tieri et al., 2018). In these review papers, VR was typically described as a “*computer-generated interactive virtual world that simulates the real world*”, a definition that applies to many rehabilitation systems. Indeed, reviews often described multiple types of mixed reality systems. Moreover, we found large differences between systems labelled as “VR” in the level of immersion, the extent to which real-world and virtual information were mixed and the type of input devices used. These broad VR definitions may not be ideal as different systems may offer different opportunities, but likely face different challenges for post-stroke rehabilitation (Garrett et al., 2018; Iosa et al., 2012; Perez-Marcos, 2018; Tieri et al., 2018). Moreover, our systematic review revealed that few reviews discuss virtual reality post-stroke cognitive rehabilitation compared to post-stroke motor rehabilitation.

### Efficacy and feasibility of VR stroke rehabilitation depends on the specific system

First, the viewing medium could influence the feasibility and efficacy of the rehabilitation. For instance, cybersickness has often been considered a contra-indication to use HMDs (Melo et al., 2018). Although studies using recent HMDs in older adults and stroke patients reported minimal cybersickness (Appel et al., 2020; Huygelier et al., 2019, 2020; Lee et al., 2020; Spreij et al., 2020), it is also known that cybersickness depends on several features of the IVR application (Davis et al., 2015; Porcino et al., 2017; Stanney & Hash, 1998; Weech et al., 2019) and end-users (Arns & Cerney, 2005).

Additionally, the viewing medium can affect multiple motor and cognitive aspects that could be relevant for rehabilitation. For instance, viewing a 3D environment with a HMD (40° horizontal field of view) reduced upper limb movement precision of healthy controls and stroke patients compared to when viewing the environment on a screen with polarizing glasses (Subramanian & Levin, 2011). Another study found that viewing a 2.5D environment (i.e., flat objects stacked behind each other) using shutter glasses improved reaching in-depth in neurological patients (van den Hoogen et al., 2012). Moreover, stroke patients and healthy controls experienced more body-ownership and presence when viewing a virtual body in a first-person perspective using a HMD than in a third-person perspective on a regular screen (Borrego et al., 2019). Another study found that navigation in a 3D environment was better when the environment was viewed with stereo-glasses versus on a 2D screen (Slobounov et al., 2015). The viewing medium may also matter for cognitive assessment and rehabilitation. For instance, one study showed that older adults performed worse in a virtual shopping memory task when assessed with an HMD than with a regular 2D computer monitor, while such effect of the viewing medium was not found for younger adults (Plechatá et al., 2019).

The design of the VR environment and visual feedback can be important too. Laver et al. (2012) stated that VR therapies to improve arm function, walking speed or independence in daily life specifically designed for the end-user group had better therapeutic effects than commercially available videogames. Moreover, the methods that are currently used to visualize patients’ movements vary widely across different systems (dos Santos et al., 2016). Some studies use AVR systems in which the patients view their own body, while other studies use SIVR systems in which a virtual avatar represents patients’ movements. However, few studies have directly compared the effects of these visualization methods on the feasibility and efficacy of therapy (dos Santos et al., 2016).

Finally, the device used to interact with the system may also influence efficacy and feasibility (Augstein & Neumayr, 2019; Milgram & Kishino, 1994; Tieri et al., 2018). Indeed, human-computer interaction studies have shown differences in usability of different input devices (Armbrüster et al., 2007; Bobeth et al., 2014; Chaparro et al., 1999; Gerling et al., 2013). Many rehabilitation systems used a motion-capture camera or wearable device as interaction device. These devices may be easier to use than handheld controllers. Indeed, some researchers stated that post-stroke rehabilitation games should be designed so that they can be played without patients using their hands (Alankus et al., 2010). However, this may not generalize to all handheld devices as one study found that stroke patients and healthy controls reported that it was easier to navigate a virtual maze using a handheld device that provided haptic feedback than when using a motion-tracking camera (Ramírez-Fernández et al., 2015). In sum, the specific devices and design of the mixed reality system are important to consider when evaluating the efficacy and feasibility of post-stroke rehabilitation systems. To facilitate conceptual clarity in post-stroke rehabilitation research, we propose new terminology to describe mixed reality systems.

### A taxonomy of mixed reality rehabilitation systems

In older definitions, immersion or presence were key features of VR (Milgram & Kishino, 1994; Steuer, 1992). Recently, Tieri et al. (2018) advocated to restrict the definition of VR to refer to the most immersive systems that use a HMD or CAVE. However, since it has been so common to use the term “VR” to refer to SIVR systems, it may be more feasible to develop more specific terms to describe different systems. Indeed, others have suggested three categories: non-immersive (i.e., desktop monitor), semi-immersive (i.e., large screen monitor or projection with more than 60° field of view), and fully immersive VR (i.e., 360° display) (dos Santos et al., 2016; Kalawsky, 1996). However, given the importance of the input device and the large differences regarding input devices between systems labelled as “VR”, we suggest a more refined classification by extending the mixed reality continuum of Milgram (Milgram et al., 1995; Milgram & Kishino, 1994). This new classification of VR rehabilitation systems can provide a basis to standardize how researchers label their VR systems. In addition, given the broad definitions of VR in our field, it is important that the specific subtype of VR is more clearly labelled in the title and abstract of a paper. Note that, as we only included reviews that specifically mentioned the term “virtual reality”, our taxonomy only reflects the diversity in systems that were labelled as “virtual reality” and does not reflect all mixed reality systems used in post-stroke rehabilitation. Thus, our taxonomy is not necessarily exhaustive and new categories can be added over time. Moreover, there may still be considerable variability between systems within a single category. However, it can be an important step towards more conceptual clarity regarding VR technology in stroke rehabilitation.

### The need to unify VR terminology within and across research domains

Although the current review focused on VR terminology within post-stroke rehabilitation, the findings have wider implications. VR has indeed received great interest in other domains, such as VR exposure therapy for phobias (Botella et al., 2017; Oing & Prescott, 2018), “*virtual anaesthesia*” (Wiederhold & Wiederhold, 2007), as biofeedback training for anxiety (van Rooij et al., 2016), as a tool to train social cognitive skills in children with autism spectrum disorders (Didehbani et al., 2016) or in combination with exercise equipment to alleviate depression (Zeng et al., 2018). Since that it is likely that the efficacy of such VR interventions is mediated by the sense of presence and realism offered, it is important to clearly describe VR systems as immersive or semi-immersive within each of these domains.

Indeed, recent reviews on VR anaesthesia and VR treatments for depression clearly specified which types of VR systems were included in their review, limiting it to systems using immersive 3D displays (e.g., Chan et al., 2018; Zeng et al., 2018). However, a clear terminology has not been widely implemented (Kardong-Edgren et al., 2019). For instance, Botella et al. (2017) reviewed the efficacy of VR exposure therapy for specific phobias, but did not specify which technological systems were considered “VR”. In addition, the reviews by Zeng et al. (2018) and Chan et al. (2018) only specified the type of VR in the methods sections of their papers, rather than in the abstract or title. Moreover, there is no consensus on how to label different VR systems, neither within nor across research domains (Kardong-Edgren et al., 2019; Tieri et al., 2018). Our mixed reality taxonomy can inspire consistent terminology to describe different mixed reality systems within and across research domains and help to unify VR terminology (Kardong-Edgren et al., 2019).

## Conclusions

Our work demonstrates that VR is often not or only broadly defined, encompassing many technologies that differ regarding human-computer interaction modalities. This vague conceptualization made it unclear which mixed reality systems had been discussed in reviews. Our analysis revealed that many reviews on post-stroke rehabilitation discussed AVR video capture and SIVR, but very few discussed IVR systems. Moreover, few reviews focused on a specific type of mixed reality. Finally, our review informed a new data-driven taxonomy of mixed reality systems, which is expected to facilitate the communication amongst researchers and clinicians working with virtual reality.

## Data Accessibility Statement

The dataset supporting the conclusions of this article is available on figshare, *https://doi.org/10.6084/m9.figshare.11902548.v1, https://figshare.com/articles/Dataset/11902548*.

The scripts used to analyze the data are available on figshare, *https://doi.org/10.6084/m9.figshare.11902554.v1, https://figshare.com/articles/Data-analyses_scripts/11902554*.

## Additional Files

The additional files for this article can be found as follows:

10.5334/pb.1033.s1 Table S1. Description and example(s) of input devices.

10.5334/pb.1033.s2 Table S2. Description of output devices.
