# Supplementary material for: The Use of the Term Virtual Reality in Post-Stroke Rehabilitation: A Scoping Review and Commentary
Source: Psychol Belg. 2021 Jun 3;61(1):145–62. doi: 10.5334/pb.1033 (PMC8176935; doi:10.5334/pb.1033)
Supplement: Table S1. — Description and example(s) of input devices. [file pb-61-1-1033-s1.pdf]

Table S1. Description and example(s) of input devices

Device	Description	Example(s)
Motion sensing handheld controller	A handheld controller that senses the motion of hand movements.	Wii remote controller
Haptic device	A motorized device that applies force feedback on the hand of the user, allowing them to feel virtual objects.	Phantom Premium Haptic Devices, Geomagic touch, Novint Falcon
Gamepad	A handheld device with a number of buttons and omnidirectional control sticks often used in video game consoles. Some gamepads include an internal motor to provide haptic feedback.	Nintendo Switch Pro Controller, Xbox controller
Motion capture wearables	A device attached to the body that can sense motion of a body part.	
Data glove	An input device worn like a glove. The global position and rotation of the glove can be tracked using a magnetic or inertial tracking device. Some data gloves can provide haptic feedback.	
Robotic device (end-effector or exoskeleton)	A *programmable* machine capable of adapting to and acting upon it's environment thus extending human abilities or replacing them in some activities(Robertson et al., 2010).	Rutgers Ankle Rehabilitation System
Force plates	A platform that measures the pressure of the user standing or moving on it.	Wii balance board
Treadmill	A device for walking or running while staying in the same place.	
Exercise bicycle	Input machine in which users push on two pedals similar to a bicycle.	Eloton SimCycle Virtual Cycling system
Motion capture camera	Sensor device that enables to use hand, finger or body movements to interact with the environment without physical contact. Motion capture cameras often only record movements of the person and not the visual appearance of the person.	Leap Motion, Microsoft Kinect, PlayStation EyeToy
Eye tracker	Sensor device that can track eye movements without physical contact.	
Touchscreen	A user can provide input to a screen using simple or complex touch gestures using a stylus or their fingers.	
Steering wheel and pedals	A wheel held by the user that can be rotated to provide input to the system. Often combined with pedals that the user can push with their feet.	
Keyboard	A device with an arrangement of multiple buttons that the user can press to type text.	
Joystick	A handheld stick that can be rotated and moved in a direction to provide input to the system.	

Computer mouse	A handheld pointing device that can detect motion in a two-dimensional space.
