# Supplementary material for: The Use of the Term Virtual Reality in Post-Stroke Rehabilitation: A Scoping Review and Commentary
Source: Psychol Belg. 2021 Jun 3;61(1):145–62. doi: 10.5334/pb.1033 (PMC8176935; doi:10.5334/pb.1033)
Supplement: Table S2. — Description of output devices. [file pb-61-1-1033-s2.pdf]

Table S2. Description of output devices.

Device	Description	Examples
2D display	An output device that displays graphical information subtending a limited field of view.	Computer monitor, TV screen
Stereo display	A display that presents offset images separately to the left and right eye to create 3D perception.	
Smart glasses	Wearable glasses that can superimpose information on top of what the user sees through the glasses.	
3D glasses	A pair of glasses worn by the user which provide an illusion of depth. In shutter glasses, images are presented separately for each eye by quickly alternating the presented image to each eye. In polarized 3D glasses, each eye sees a different image due to the use of polarizing filters on the glasses.	Crystal Eyes CE-2
Cave automatic virtual environment	A virtual environment is projected on 3 to 6 walls of a room or large displays surrounding the user.	
Head-mounted display	A stereoscopic display device worn on the head.	Sony Glasstron
VR Headset	A head-mounted device comprising of a stereoscopic display, stereo sound and head motion tracking sensors. Some VR headsets include eye trackers and game controllers.	Oculus Rift, HTC Vive
